# Sharpening the Edge for Precision Cancer Immunotherapy: Targeting Tumor Antigens through Oncolytic Vaccines

**DOI:** 10.3389/fimmu.2017.00800

**Published:** 2017-07-13

**Authors:** Namit Holay, Youra Kim, Patrick Lee, Shashi Gujar

**Affiliations:** ^1^Department of Pathology, Dalhousie University, Halifax, NS, Canada; ^2^Department of Microbiology and Immunology, Dalhousie University, Halifax, NS, Canada; ^3^Department of Biology, Dalhousie University, Halifax, NS, Canada; ^4^Centre for Innovative and Collaborative Health Sciences Research, Quality and System Performance, IWK Health Centre, Halifax, NS, Canada

**Keywords:** cancer immunotherapy, oncolytic vaccines, tumor antigens, antitumor immunity, T cells, tumor major histocompatibility complex ligandome

## Abstract

Cancer immunotherapy represents a promising, modern-age option for treatment of cancers. Among the many immunotherapies being developed, oncolytic viruses (OVs) are slowly moving to the forefront of potential clinical therapeutic agents, especially considering the fact that the first oncolytic virus was recently approved by the Food and Drug Administration for the treatment of melanoma. OVs were originally discovered for their ability to kill cancer cells, but they have emerged as unconventional cancer immunotherapeutics due to their ability to activate a long-term antitumor immune response. This immune response not only eliminates cancer cells but also offers potential for preventing cancer recurrence. A fundamental requirement for the generation of such a strong antitumor T cell response is the recognition of an immunogenic tumor antigen by the antitumor T cell. Several tumor antigens capable of activating these antitumor T cells have been identified and are now being expressed through genetically engineered OVs to potentiate antitumor immunity. With the emergence of novel technologies for identifying tumor antigens and immunogenic epitopes in a myriad of cancers, design of “oncolytic vaccines” expressing highly specific tumor antigens provides a great strategy for targeting tumors. Here, we highlight the various OVs engineered to target tumor antigens and discuss multiple studies and strategies used to develop oncolytic vaccine regimens. We also contend how, going forward, a combination of technologies for identifying novel immunogenic tumor antigens and rational design of oncolytic vaccines will pave the way for the next generation of clinically efficacious cancer immunotherapies.

## Introduction

Immunotherapies have steadily emerged as a powerful treatment option for patients with various types of cancers. While employing the immune system to fight cancer was first proposed in the late nineteenth century, it was only recently that the improved understanding and novel discoveries in classical and tumor immunology have led to the design of more targeted and efficacious immunotherapeutics (1–4). One such immunotherapy utilizes oncolytic viruses (OVs), which were originally discovered for their direct cancer-killing properties (5). Historically, case reports of cancer regression following infection with unrelated viruses started appearing around the early twentieth century (6). But it was not until the 1990s that concrete evidence demonstrated the ability of certain viruses to preferentially target cancer cells (7–9). In recent times, the advent of new technologies allowing for customization of viruses, combined with the urgent need for novel and effective therapies for cancer treatment, has led to a new impetus for OV research (10). OVs have a dual mechanism of action against tumors. First, they can preferentially replicate in and directly kill cancer cells, in a mechanism known as oncolysis (11). Second, the immunological events induced following the administration of OVs awaken the previously suppressed immune system to become activated and target tumor cells more effectively (12). This activation of the immune system is the most promising aspect of oncolytic virotherapy. One of the major players of the immune system responsible for targeting cancer cells are T cells and the effective activation of tumor-specific T cells can lead to long-term antitumor immunity and protection against cancer recurrence (13).

For the activation of antitumor T cells, the primary requirement is the presentation of a tumor antigen *via* major histocompatibility complex (MHC) molecules of antigen-presenting cells (APCs) (14). Antigens, usually identified as small peptide molecules of approximately 8–18 amino acids in length, are expressed *via* MHC class I and II molecules, and lead to the activation of antigen-specific CD8 and CD4 T cells, respectively (15). Tumor antigens can be derived from peptide fragments of mutated oncoproteins and tumor suppressors, aberrantly expressed cellular proteins, modified glycoproteins, oncofetal proteins, tissue-specific differentiation proteins, and proteins derived from oncogenic viruses (16, 17). Identification of such tumor antigens to activate antigen-specific T cell responses in tumors represent a highly attractive target for cancer immunotherapies today (16, 18). In addition to the antigenic peptide presented through the MHC molecule, the complete activation of T cells requires two other signals: costimulatory molecules on APCs and the presence of the appropriate cytokines in the immune milieu (19, 20). Thus, ongoing research to improve cancer immunotherapies aim to target one or more of these signals to effectively stimulate clinically relevant antitumor T cells.

In this mini-review, we highlight the studies that have incorporated tumor antigens in OVs to enhance antitumor immune responses and consequent therapeutic benefits in the context of cancer. We discuss recent studies completed using a variety of viral systems, as well as combinations of multiple strategies used to elicit the most efficacious immune response. We also throw light on some of the challenges in this area of research and emphasize the need for combining recent, cutting-edge technologies for tumor antigen discovery with oncolytic virus research for generating more efficacious cancer treatments.

## Oncolytic Vaccine Therapy

The first generation of OVs primarily focused on direct killing of tumor cells. OVs can replicate preferentially in tumor cells due to deregulated signaling pathways (8, 9, 21, 22) resulting in increased susceptibility of tumor cells to viral infections (22–24). When it was observed that the direct killing of tumor cells led to the release of novel tumor antigens in the tumor microenvironment and the subsequent activation of immune responses (25–29), strategies began to be focused on the modulation and optimization of these immune responses to achieve maximum clinical benefit. The overexpression of tumor antigens *via* OVs represents one such strategy that makes OV-based cancer therapies more potent by driving immune responses to be directed specifically toward the tumor. OVs that are genetically modified to express tumor antigens are commonly known as “oncolytic vaccines.”

### Vesicular Stomatitis Virus (VSV)

In recent times, many OVs have been found to be amenable for therapeutically desired genetic modifications. Among these, VSV has been the subject of extensive genetic manipulation and consequent investigation on antitumor immunity in the context of cancer treatment. For example, studies have shown that VSV-expressing tumor antigens human papilloma virus oncogene E7 (VSV-E7) and human dopachrome tautomerase (VSV-hDCT) can induce tumor antigen-specific CD8 cytotoxic T cell responses (30, 31). Therapeutic vaccination with VSV-E7 led to reduced TC-1 tumor volumes, and VSV-hDCT generated antigen-specific CD4 T cell responses in addition to CD8 T cells in murine melanoma (30, 31). Another study employed the popularly used ovalbumin (ova) as a surrogate tumor antigen expressed in murine melanoma cells to demonstrate that the administration of VSV engineered to express ova (VSV-ova) led to increased activation of naïve T cells, as well as increased number of ova-specific, antitumor T cells (32).

Furthermore, VSV-based oncolytic vaccines have also been indicated as promising candidates to be used in combination therapies. A recent study demonstrated that in combination with stereotactic ablative radiation therapy, VSV-ova helped control local and systemic disease in a murine oligometastatic melanoma model (33). In addition, oncolytic vaccines can be administered in combination with adoptive transfer of antigen-specific T cells for enhanced therapeutic benefits compared to either treatment alone. For instance, a melanoma-derived tumor antigen gp100-expressing VSV (VSV-gp100) combined with adoptive transfer of gp100-specific T cells resulted in increased survival of mice with established melanoma that was accompanied by the development of antitumor T cell responses (34, 35). Similar results were also observed by combining VSV-ova and adoptive transfer of ova-specific T cells (32). Taken together, these studies in preclinical models demonstrated that oncolytic vaccines may be combined with current clinical treatment options to achieve improved therapeutic and immune responses.

Another interesting approach to develop efficacious VSV-based oncolytic vaccines was to employ tumor-derived cDNA libraries. Specifically, cDNA libraries derived from cancer cell lines are expressed in VSV, followed by screening and administration of these library-based oncolytic vaccines in tumor-bearing mice. VSV expressing a cDNA library created from normal prostate cells has been shown to lead to the rejection of mouse prostate cancers with little autoimmunity as measured by whitening of whiskers and tail, hair depigmentation, abnormal immune cell infiltration, and tissue destruction (36). A subsequent study employed VSV to express a melanoma cDNA library, which was screened *in vitro* for immunogenic tumor antigens, and demonstrated that a combination of three specific VSV-cDNA viruses infected established melanoma tumors and induced tumor rejection *via* T_H_-17 responses (37).

These studies using cDNA library-expressing VSV also highlighted the importance of treating cancers according to their origins. First, it was observed that primary and recurring tumors must be targeted in a different manner and thus, “recurrence” libraries were developed. In one study employing a murine melanoma model, 14 out of 16 recurrences were found to have mutated BRAF, so VSV-BRAF was used to successfully target the recurring tumors (38). Second, the anatomical site of cancer development is another important consideration for oncolytic vaccine design. For example, a combination of VSV viruses derived from the melanoma cDNA library (VSV-N-RAS, VSV-CYTC-C, and VSV-TYRP-1) that was successful in treating subcutaneous melanoma could not treat intracranial melanoma tumors. Instead, a therapeutic combination targeting new tumor antigens in the context of intracranial tumors (VSV-HIF-2α, VSV-SOX-10, VSV-C-MYC, and VSV-TYRP-1) was shown to promote long-term survival of mice with intracranial melanoma (39). Building on this, another study showed that tumors of different histological origin shared immunological signatures based on their location and could be targeted specifically (40). The study used a glioma model in comparison to the intracranial melanoma model to establish that different tumors growing in the same location shared location-specific immunological signatures that could be targeted with the right combination of oncolytic vaccines. Of note, this study was among a few that combined oncolytic vaccines with immunological checkpoint inhibitors (ICIs) to reveal that ICIs enhanced the impact of oncolytic vaccines by reactivating T_H_-1 and T_H_-17 responses. Using ICIs like anti-PD1 and anti-CTLA4 antibodies in combination with OVs represents the next frontier in cancer immunotherapy as these complementary therapies are emerging as synergistic therapeutic partners of each other (41–44). Going forward, identification of novel tumor antigens (discussed in Section “Future directions and concluding remarks”) for expression in viral vectors combined with effective combination therapy *via* ICIs represents an emerging paradigm of cancer immunotherapy.

### Vaccinia Virus (VV)

Another virus that has allowed for ease of genetic manipulation and, thus, lent itself to oncolytic vaccine research is VV. VV has been employed in multiple studies for prophylactic vaccine development. Studies using oncofetal tumor antigen animal models like carcinoembryonic antigen (CEA) and glycoprotein oncofetal tumor antigen (5T4) transgenic mice have been widely used to study the prophylactic capacity of VV vectors. One such study demonstrated that VV-expressing CEA administered in CEA transgenic mice led to the development of CEA-specific T_H_-1 responses and peptide-specific cytotoxicity. The authors noted that neither CEA antibodies nor CEA-specific T cell responses were elicited in CEA transgenic mice in response to endogenous or administered CEA in the absence of virus, indicating the usability of this animal model for more aggressive vaccinations (45). Upon virus infection, protection against CEA-specific cancer cells was observed without any effect on normal tissue-expressing CEA (45). Another study employing VV-expressing human and mouse 5T4 (VV-h5T4, VV-m5T4) demonstrated that mice vaccinated with these vectors showed retarded tumor growth upon challenge with syngeneic melanoma and colorectal cancer cells (46). No autoimmune toxicity in the form of wasting, respiratory problems, affected mobility or weight loss was seen in this study. Put together, these studies reaffirmed the potential of oncolytic vaccines as safe therapeutic options for treatment of cancers with low off-site toxicity.

Engineering genetic elements other than tumor antigens for immunomodulation is a common strategy for developing viral vectors. VV has provided an excellent platform for engineering costimulatory elements in conjunction with tumor antigens to incorporate other important factors for T cell activation. One such study demonstrated the expression of a T cell engager (TCE) element along with tumor antigen EphA2 in a VV vector (47). The TCE is a special secretory element used to specifically bind and activate T cells *via* CD3. The study noted that the virus killed tumor cells and induced a bystander killing of non-infected tumor cells *in vitro*. The modified VV also had potent antitumor activity *in vivo* in a lung cancer xenograft model (47). Another study employed VV to express a triad of costimulatory molecules (B7-1, ICAM-1, and LFA-3) along with oncofetal tumor antigen CEA, and the administration of the modified virus increased survival of colon adenocarcinoma tumor-bearing mice due to induced CD8 and CD4 T cell responses. Clinical serum and urine assays combined with histopathology showed no classical indicators of autoimmune responses, a typical complication when targeting oncofetal antigens like CEA (48).

The final factor that determines the quality of the generated T cell responses is the presence of cytokines in the immune environment. Expressing cytokines *via* viral vectors represents a promising strategy for the development of a robust antitumor immune response, and thus far, many cytokines including IL-2, TNF, IFN, and GM-CSF have been expressed through OVs (49–52). In 2015, the U.S. Food and Drug Administration approved the first oncolytic virus T-VEC for use in clinics. T-VEC employs modified herpes simplex virus (HSV)-expressing GM-CSF to enhance the generation of APCs (53). In the context of oncolytic vaccines and cytokines, a study using MB49 cancer cells expressing male tumor antigen HY was used to evaluate the efficacy of VV-overexpressing HY (VV-HY) and GM-CSF (VV-GM-CSF) to overcome tumor-associated immune tolerance. The study noted that the administration of both viruses together led to the generation of splenic HY-specific CD8 T cells, indicating development of systemic immunity (54). Overall, consideration of all the signals required for an effective T cell response is an important parameter for future studies employing oncolytic vaccines to activate holistic antitumor responses that are qualitatively superior to current therapeutic regimens.

### Other Viruses

Several other viruses have been employed as oncolytic vaccines to target tumor antigens. A unique study worth highlighting employed Sindbis virus engineered with β-galactosidase (SV-β-gal) to demonstrate memory T cell responses that conferred protection against tumor re-challenge with antigen-specific and non-specific colon cancer cells in mice (55). The authors demonstrated that the influx of NKG2D-expressing antigen-specific CD8 T cells in the tumor was important for the development of long-lasting memory responses (55). Another study compared homologous vaccination strategies with Semliki Forest virus, adenovirus, and pox virus, and found that Semliki Forest virus provided potent protection in P185 tumors and showed increased levels of systemic antitumor-specific central memory T cells (56). However, the role of memory T cell development upon oncolytic vaccine administration remains largely unknown and poorly characterized. Considering the importance of antitumor T cell memory responses in long-term protection, the focus of future studies aimed at dissecting the immunological implications of oncolytic vaccines should consider memory T cells.

Other viruses that have been employed to target tumor antigens include Newcastle disease virus (NDV) and HSV. A study using NDV-expressing β-galactosidase-derived antigenic peptide (NDV-β-gal) found enhanced antitumor immune responses in a murine colon cancer model (57). The importance of cytokines in enhancing antitumor T cell responses has been discussed earlier. This study also demonstrated that coadministration of NDV-β-gal with NDV-expressing IL-2 led to increased frequency of tumor-infiltrating antigen-specific T cells and enhanced tumor regression (57). In another study, HSV was employed to express xenoantigen prostatic acid phosphatase (HSV-hPAP). This study demonstrated that HSV-hPAP caused reduced tumor growth and increased survival in mice bearing prostate tumors (58). A complete list of studies employing OVs to target tumor antigens has been summarized in Table 1.

**Table 1:** Summary of oncolytic vaccine studies using a variety of viral vectors targeting respective tumor antigens.

Antigen	Implementation strategy	Route of delivery	Physiological effect	Reference
**Vesicular stomatitis virus**				
E7	Monotherapy	Intramuscular	Antigen-specific CD8 T cell responses	(30)
			Tumor volume reduction	
DCT	Monotherapy	Intranasal	Antigen-specific CD8 and CD4 T cell responses	(31)
DCT	Heterologous prime-boost	Intranasal	Increased antigen-specific T cells	(31)
			Enhanced prophylactic and therapeutic efficacy	
Ova	Monotherapy	Intratumoral	Increased T cell activation	(32)
			Increased antigen-specific T cells	
Ova	Combination therapy	Intravenous	Local and systemic disease control	(33)
gp100	Combination therapy with adoptive transfer	Intratumoral	Increased antigen-specific T cells	(34, 35)
			Elimination of established tumors	
Various	Viral expression of cDNA libraries	Intravenous	Tumor rejection *via* CD4 T_H_-17 responses	(36–40)
			Anatomy-specific immune signatures of tumors	
gp33	Novel delivery approach	Multiple	Oncolytic vaccine delivery using B cells	(62)
**Vaccinia virus**				
CEA	Monotherapy	Subcutaneous	Antigen-specific CD4 T cell responses	(45)
			Peptide-specific cytotoxicity	
			No autoimmune responses	
CEA	Engineered with costimulatory elements	Intravenous	Activation of CD4 and CD8 T cells	(48)
			Increased survival	
5T4	Monotherapy	Intravenous/intramuscular	Retarded tumor growth	(46)
			No autoimmune responses	
Ova	Heterologous prime-boost	Intraperitoneal	Increased antitumor activity	(60)
			Antigen-specific CD8 T cell responses	
E7	Heterologous prime-boost	Intraperitoneal	Antigen-specific T cell responses	(61)
HY	Combination therapy	Intratumoral	Systemic antigen-specific CD8 T cell responses	(54)
EphA2	Engineered with T cell engager element	Intraperitoneal	Direct killing of cancer cells	(47)
			Bystander killing of cancer cells	
gp33	Novel delivery approach	Multiple	Oncolytic vaccine delivery using B cells	(62)
**Adenovirus**				
DCT	Heterologous prime-boost	Intravenous	Antigen-specific T cell responses	(59)
			Increased survival	
DCT	Heterologous prime-boost	Intramuscular	Increased antigen-specific T cells	(31)
			Enhanced prophylactic and therapeutic efficacy	
Trap1a	Heterologous prime-boost	Intradermal	Effective tumor protection	(56)
			Increased CD8 T cell responses	
gp33	Novel delivery approach	Multiple	Oncolytic vaccine delivery using B cells	(62)
**Newcastle disease virus**				
β-gal	Combination therapy with NDV-IL-2	Intratumoral	Increased tumor regression	(57)
			Increased antigen-specific TILs frequency	
**Herpes simplex virus**				
PAP	Monotherapy	Intravenous	Reduced tumor growth	(58)
			Increased survival	
**Sindbis virus**				
β-gal	Monotherapy	Intraperitoneal	Memory T cell responses	(55)
			Antigen-specific and non-specific immunity	
E7	Heterologous prime-boost	Intramuscular	Antigen-specific T cell responses	(61)
**Semliki Forest virus**				
Trap1a	Homologous injections	Intradermal	Increased tumor-specific central memory	(56)
Trap1a	Heterologous prime-boost	Intradermal	Effective tumor protection	(56)
			Increased CD8 T cell responses	
Ova	Heterologous prime-boost	Intraperitoneal	Increased antitumor activity	(60)
			Antigen-specific CD8 T cell responses	
**Maraba virus**				
DCT	Heterologous prime-boost	Intravenous	Antigen-specific T cell responses	(59)
			Enhanced survival of mice	
**Pox virus**				
Trap1a	Heterologous prime-boost	Intradermal	Effective tumor protection	(56)
			Increased CD8 T cell responses	

*DCT, dopachrome tautomerase; Ova, ovalbumin; gp100, glycoprotein 100; gp-33, lymphocytic choriomeningitis virus-derived peptide; CEA, carcinoembryonic antigen; 5T4, glycoprotein oncofetal tumor antigen; EphA2, Ephrin type-A receptor 2; Trap1a, tumor rejection antigen P1A; β-gal, β-galactosidase; PAP, prostatic acid phosphatase*.

## Heterologous Virus Prime-Boost Strategies

One of the major challenges that oncolytic vaccines face is the induction of an undesired antiviral immune response against the viral vector. This antiviral immunity reduces the efficacy of the OV treatment by clearing the virus prematurely. In the context of oncolytic vaccines, immune responses against the viral vector may dominate the response against the tumor antigen, generating a much more subdued antitumor response. To overcome this obstacle, strategies that effectively redirect the immune responses against the tumor must be employed. One such strategy that we shall discuss is the heterologous virus prime-boost strategy.

The heterologous virus prime-boost strategy exploits the quick and effective immunological recall responses to redirect the maximal potential of immunity toward the tumor antigen. In the first priming step, a tumor antigen is expressed using one oncolytic virus vector, whereby the immune system “sees” and responds to the tumor and viral antigen for the first time. In the second boost step, a different viral vector is chosen to express the same tumor antigen, resulting in a primary immune response against the second viral vector while a stronger memory immune response is induced against the tumor antigen. Thus, this clever manipulation allows for the skewing of immunity toward antitumor responses over antiviral responses.

In preclinical studies, adenoviruses (Ad), VSV, and VV have emerged as common viral vectors that can be adapted for evaluation of priming and boosting strategies. Using the previously discussed tumor antigen hDCT, studies have shown that priming with Ad-hDCT and boosting with Maraba virus-expressing hDCT led to increased hDCT-specific T cell responses and survival of melanoma tumor-bearing mice (59). Another study using the same tumor antigen in a murine melanoma model demonstrated that priming with VSV-hDCT followed by a booster with Ad-hDCT also greatly enhanced antigen-specific T cell responses and led to enhanced efficacy in a prophylactic as well as a therapeutic setting (31). This study also demonstrated that VSV could boost hDCT-specific T cell responses generated by Ad-hDCT priming, indicating that VSV could successfully be used to either prime or boost tumor T cell responses (31). Another study demonstrating that the prime-boost strategy is effective irrespective of the order in which certain versatile viral vectors are administered used VV and Semliki Forest virus both overexpressing ova, resulting in enhanced antitumor activity and increased levels of ova-specific CD8 T cells in a murine ovarian surface epithelial carcinoma animal model (60). VV has also been used in combination with Sindbis virus in TC-1 tumor models for the prime-boost strategy, where priming with Sindbis-E7 and boosting with VV-E7 resulted in effective antitumor immune responses and the generation of increased numbers of E7-specific CD8 T cells (61).

Unfortunately, one of the major challenges with the heterologous virus prime-boost strategy involves the rapid killing of APCs by effector T cells before the memory T cells are engaged to produce a strong antitumor T cell response. A unique study with oncolytic vaccines addressed this issue by delivering oncolytic vaccines with B cells. Here, the immunodominant gp33 peptide derived from lymphocytic choriomeningitis virus was expressed in three different viruses VSV, Ad, and VV to demonstrate that B cells loaded with the booster virus can elicit better antigen-specific secondary T cell responses, which were dependent on antigen presentation by dendritic cells (62).

Altogether, the heterologous virus prime-boost strategy represents an innovative approach to redirecting immune responses in oncolytic vaccine therapy and warrants further research in the areas of viral vector selection and delivery of vaccines. These strategies combined with effective oncolytic vaccine design and vector development can help maximize antitumor immune responses and the development of efficacious clinical regimens.

## Challenges for Oncolytic Vaccine Therapy

Oncolytic vaccine development has come a long way from its initial stages but still has ways to go. As alluded to earlier, implementation of strategies like the heterologous virus prime-boost warrants more research. An important factor when extending such strategies to clinics is a consideration of pre-existing immunity to common viral vectors, which may lead to reduced efficacy in humans. Understanding pre-existing immunity combined with choosing the most effective routes of delivery, which depends on the type of tumor being targeted, will help achieve maximum efficacy of oncolytic vaccines in clinics. Routes of delivery employed in the reviewed studies, along with other parameters and findings, have been summarized in Table 1. More research on developing the criteria for matching tumor type with routes of delivery is much needed for effective translation of preclinical models to bedside treatments. Moreover as discussed above, development of autoimmunity upon implementation of oncolytic vaccines has been a long-standing scientific concern. Minimizing autoimmune responses and maximizing tumor-specific T cell responses needs to be a major focus of future studies aimed at developing oncolytic vaccines. In this context, choice of tumor antigen can play an important role in not only maximizing antitumor immunity but also reducing off-site toxicity if carefully selected. In light of ongoing burgeoning developments in the areas of cancer genomics and proteomics, it is believed that novel antigens that are highly tumor-specific, and thus are non-autoreactive, will be available for such targeting (63, 64). In the following section, we discuss how, going forward, novel tumor antigens discovered *via* a combination of computational and high throughput approaches provide the best chance for selecting antigens to be targeted in tumors and represent an emerging frontier in cancer immunotherapy.

## Future Directions and Concluding Remarks

Novel tumor antigen discovery is a fundamental cornerstone in cancer immunotherapy and provides an enormous knowledge base for oncolytic vaccine research to draw from. Cutting-edge technological advances in tumor antigen discovery have identified a wide range of potential targets that can be incorporated in the next generation of oncolytic vaccines for designing highly targeted and efficacious therapies. One of these major advances is in the field of sequencing; new and improved sequencing technologies are now available to identify and categorize mutations at the level of genes, exons, and RNA from many different cancer types (65–67). In addition, recent developments in computational techniques allow better *in silico* prediction and identification of immunogenic tumor antigens (68–74). Finally, advances in peptide isolation and identification by immunoprecipitation and mass spectrometry-based proteomics, respectively, have led to the development of novel approaches to unearth new tumor antigens with greater precision (75–77). One such result of modern research combining these advances in different fields has led to the discovery of the high-confidence tumor MHC ligandome, which represents an array of tumor-derived peptide antigens that are bound to MHC molecules. These tumor antigens are identified using immunoprecipitation of peptide-MHC molecules followed by mass spectrometry analysis of the peptides eluted from the MHC molecules. Such antigens have been collectively called as ligandome-derived tumor-associated antigens (LiTAAs) (78–81). A recent breakthrough study used a combination of exome sequencing, proteomics, and computational modeling to identify novel tumor antigens that could be targeted *via* peptide-based vaccines (82). Going forward, the tumor MHC ligandome will serve as a unique and effective library for identifying novel tumor antigens to be targeted using oncolytic vaccines. More importantly, as opposed to the traditional peptide-based vaccines to deliver LiTAAs, oncolytic vaccines provide the advantage of administering LiTAAs in the presence of all the necessary immune signals for the proper activation of long-term antitumor immunity.

**Figure 1:**
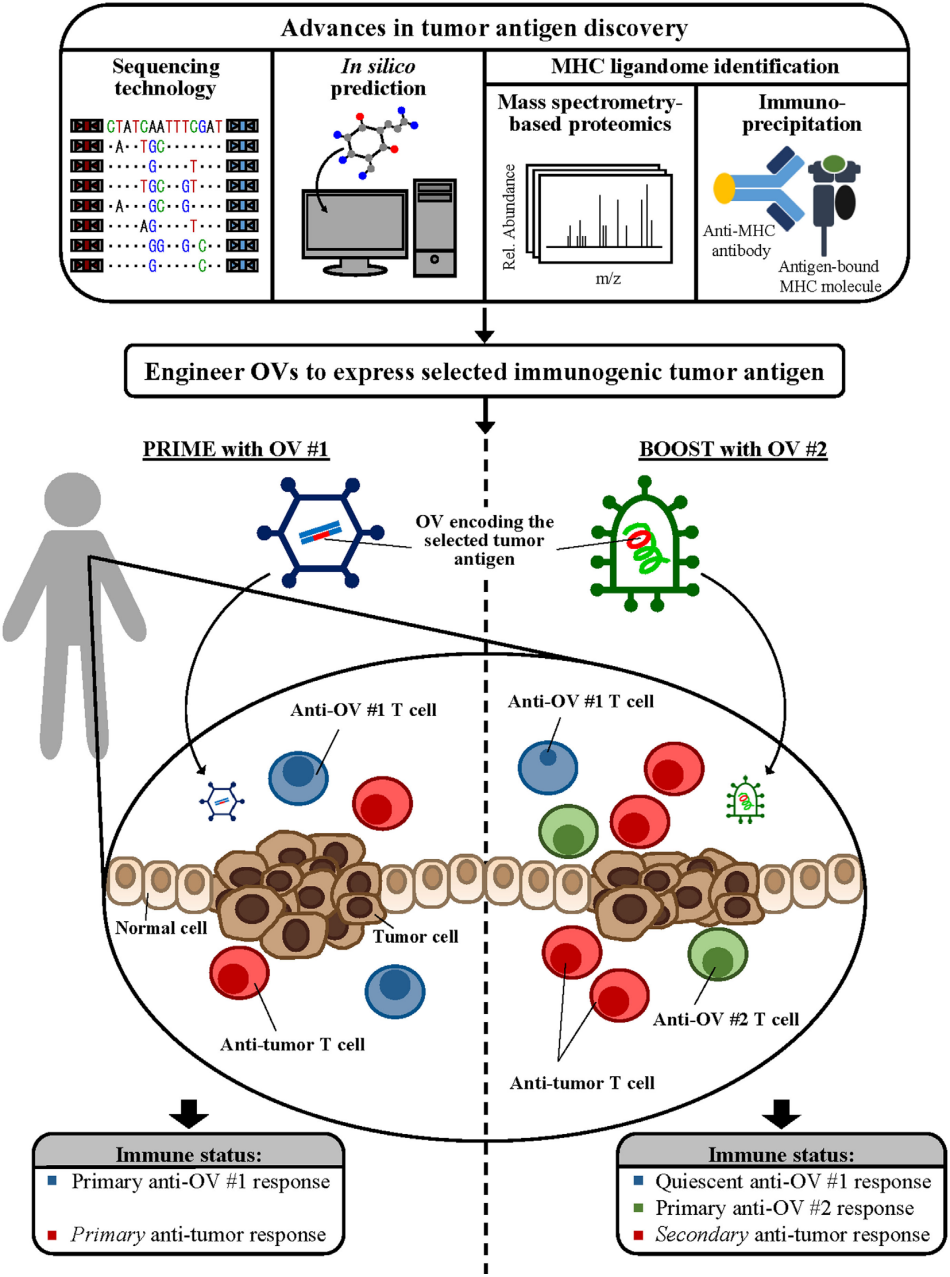
Combining novel antigen discovery platforms to design oncolytic vaccines. Advances in sequencing technology, *in silico* prediction methods, and mass spectrometry-based proteomics and immunoprecipitation for major histocompatibility complex (MHC) ligandome elucidation allow identification of novel tumor antigen targets. Engineering these novel antigens into powerful viral vectors will provide a platform for the development of the next generation of oncolytic vaccines. Incorporating immunomodulatory strategies, such as the heterologous virus prime-boost as shown, during oncolytic vaccine administration can maximize antitumor immune responses, leading to the development of complete and clinically efficacious antitumor treatment options.

Combining this unique capability of oncolytic vaccines with novel tumor antigen discovery paradigms and effective, timely immunological intervention *via* strategies like the heterologous virus prime-boost will aid the design of the most clinically efficacious cancer immunotherapeutics (summarized in Figure 1). With the ever-increasing knowledge bases that have not yet been effectively translated to the bedside, concerted efforts to adapt these uniquely exciting strategies to develop therapeutic models of high clinical relevance are more important today than ever before and mark the next frontier in precision cancer immunotherapy research.

## Author Contributions

NH, YK, and SG developed the concept. NH prepared the figures and wrote the manuscript. YK participated in writing and technical editing of the manuscript. PL and SG supervised and edited the manuscript.

## Conflict of Interest Statement

The authors declare that the research was conducted in the absence of any commercial or financial relationships that could be construed as a potential conflict of interest.
